# Fertility preservation in women with cervical, endometrial or ovarian cancers

**DOI:** 10.1186/s40661-016-0029-2

**Published:** 2016-07-27

**Authors:** Michael Feichtinger, Kenny A. Rodriguez-Wallberg

**Affiliations:** 1 Department of Obstetrics and Gynecology, Division of Gynecological Endocrinology and Reproductive Medicine, Medical University of Vienna, Vienna, Austria; 2 Wunschbaby Institut Feichtinger, Vienna, Austria; 3 Department of Oncology – Pathology, Karolinska Institutet, Stockholm, Sweden; 4 Department of Obstetrics and Gynecology, Section of Reproductive Medicine, Karolinska University Hospital, Novumhuset Plan 4, SE-141 86 Stockholm, Sweden

**Keywords:** Fertility preservation, Gynecological cancer, QoL, IVF, Cervical cancer, Ovarian cancer, Endometrial cancer, Pregnancy

## Abstract

**Background:**

Although cancer in general affects an aged population, a significant number of women develop cancer at childbearing age. Long-term survival rates after gynecological cancer, especially in young patients are increasing and all quality-of-life aspects, including preservation of fertility have become of major relevance.

**Outcomes:**

Surgical techniques aimed at sparing reproductive organs and preserving fertility have been developed for women presenting with gynecological cancer found at early stages. Indications for fertility-sparing surgery are in general restricted to women presenting with a well-differentiated low-grade tumor in its early stages or with low malignant potential. Up to now, use of fertility-sparing techniques in well-selected patients has not been shown to affect overall survival negatively and fertility outcomes reported have been favorable. Still larger amounts of data and longer follow-up periods are needed. Several current fertility-sparing cancer treatments may result in sub-fertility and in those cases assisted reproductive techniques are indicated. Overall quality of life has been satisfactory in cancer patients after fertility-sparing surgery.

**Conclusions:**

Fertility-sparing surgery is a viable tool to enable gynecological cancer patients of young age to fulfill their family building without impairment of oncological outcome. Cancer patients of reproductive age should undergo fertility counseling to analyze this sensitive subject. Further studies are needed to investigate the role of fertility-sparing treatment and combined adjuvant therapy in higher-grade cancers.

## Background

The overall cancer risk in women below the age of 39 years is estimated to be one in 39 [1]. Of all gynecological cancer cases, young women comprise 2 % of cervix cancer cases, 5 % of endometrial cancers and up to approximately 12 % of ovarian cancers [1]. Five-year survival rates range from 46 % in ovarian cancer to more than 80 % in endometrial cancer and over 90 % in cases of borderline ovarian tumors [2, 3]. Infertility following cancer treatment has been recognized as a main concern as regards quality of life (QoL) in cancer patients [4, 5]. As a result of improved long-term survival rates in young people, all QoL aspects are of major importance. Additionally, due to current social trends, childbearing nowadays is delayed, hence an increasing number of women that present with cancer at a young age might have not yet fulfilled their family building plans and will be interested in undergoing treatments that would preserve their chances to have children in the future [6]. Most oncologic treatments have detrimental effects on female reproductive potential, in particular those including chemotherapy with agents of high gonadotoxicity, or radiation therapy in a field involving the ovaries, the uterus and the vagina, which may be compromised and damaged by direct irradiation [7]. The resumption of menstrual cycles indicates that some ovarian function is maintained, but it does not guarantee fertility, and early onset of menopause in women previously treated for cancer is a common finding [7–9].

Surgery is currently the most effective treatment for cancer and eventually up to 100 % of patients may be cured when complete removal of a tumor is achieved. Surgery may also be indicated for treatment of premalignant disease of the cervix or the endometrium in female patients, as cancer prophylaxis. Conization, for example, may lead to completely disease-free follow-up, but it may induce sub-fertility by affecting the normal function of the cervix and its glandular secretion. Infertility induced by such forms of intervention may be overcome by treatments involving assisted reproductive technologies, such as intrauterine insemination or in vitro fertilization, IVF.

In gynecologic oncologic surgery, there has been gradual development of fertility-sparing surgery with the aim of preserving the reproductive organs. Survival should not be compromised and thus indications are restricted to patients of a young age with a desire to preserve fertility and presenting with a well-differentiated cervical, ovarian or endometrial low-grade tumor in its early stages or with low malignant potential.

In this article we will discuss indications for fertility-sparing methods available to women with gynecological cancer, and up-to-date data on safety and efficacy as regards oncologic outcomes and reproductive outcomes including obstetric outcomes and quality of life.

### Cervical cancer

Cervical cancer makes up 1.5 % of all new cancer cases in females. In 2015, 12 900 patients with a median age of 49 years had newly diagnosed cervical cancer in the US. Of these, 38.5 % were under the age of 45 years [1, 2]. The different approaches regarding fertility-sparing surgery in cases of cervical cancer are summarized in Table 1.

**Table 1:** Fertility-sparing interventions in women with cervical or endometrial cancer

Diagnosis	Type of Surgery	Description	Reproductive and Obstetric Outcomes	Oncologic Outcome	Quality of Life
Cervical Cancer FIGO Stage IA1 (microinvasion <3 mm)	Large loop excision of the transformation zone (LLETZ) or conization if absence of lymph vascular space invasion and negative margins are confirmed	Complete resection of the transformation zone	No fertility impairment reported. OR 1.7 for preterm delivery and 2.69 for premature rupture of membranes; associated with resection size. No difference in neonatal outcome [130]	Similar oncologic outcomes reported in comparison with hysterectomy [10]	Conization has not been associated with reduced quality of life or sexual satisfaction [49]
FIGO Stages IA2, IB1 < 2 cm	Cervical conization and laparoscopic lymphadenectomy	Conization of the cervix and laparoscopic pelvic lymphadenectomy	Spontaneous conceptions of about 47 %. Prematurity rates reported with 14.3 % of infants born <32 weeks of gestation [21]	Excellent rates of 5-year disease-free survival (97 %) [21]	Conization with laparoscopic lymphadenectomy has not been associated with reduced quality of life or sexual satisfaction [49]
FIGO Stages IA2, IB1	Radical trachelectomy. Techniques described for vaginal, abdominal, laparoscopic or robotic trachelectomy	Resection of the cervix and surrounding parametria with conservation of the uterus and the ovaries, pelvic lymphadenectomy	Spontaneous pregnancy rates in >60 % of patients Preterm deliveries with 28 % of infants born <32 weeks of gestation [17, 132]	Rates of recurrence and mortality are comparable with those described for similar cases treated with radical hysterectomy; long-term survival 98.4 %. Low relapse rates (4.5 %) [16, 17]	Lower quality of life than healthy controls but similar to radical hysterectomy No significant impairment in sexual satisfaction Long-term bladder complications (40 %) and lymphedema (10 %) [46–48]
FIGO Stage IB1, >2 cm	Neoadjuvant chemotherapy followed by radical trachelectomy	Three cycles of paclitaxel, cisplatin and ifosfamide followed by radical trachelectomy	After neoadjuvant chemotherapy and trachelectomy up to 86 % live-birth rates with 86 % spontaneous conception rate [134]	Reported relapse rate of 7.6 % with 90 % survival [23, 24]	Lack of data
Endometrial Cancer FIGO stage IA	Medical conservative treatment with hormone therapy using progestational agents either orally or by IUD for >6 months Myometrial evaluation by MRI should be performed to confirm absence of myometrial infiltration and no extrauterine involvement [52].	Follow-up by hysteroscopic exams with endometrial biopsies every 3 months	Pregnancy rates of >60 % Uneventful pregnancies reported [63, 72]	Positive response rate to progesterone treatment of 72 %. Either oral or local IUD treatments proposed, as well as a combination of both. Relapse rate of 50 %. A second round of progesterone therapy in cases of relapse has been associated with a response rate of up 89 % [55, 57, 60, 62]. A levonorgestrel IUD has shown greater regression on histology, lower relapse rates and lower rates of hysterectomy for treatment of complex endometrial hyperplasia vs. oral progesterone [57–59].	Levonorgestrel IUD treatment has been associated with fewer systemic side effects compared with oral progesterone administration [79, 80]

Modified from: Rodriguez-Wallberg KA, Oktay K. Fertility preservation during cancer treatment: clinical guidelines. Cancer management and research. 2014;6:105-17

Abbreviations: *FIGO* International Federation of Gynecology and Obstetrics, *LLETZ* large loop excision of the transformation zone; *IUD* intrauterine device, *OR* odds ratio

In cases of micro-invasion (< 3 mm), FIGO stage IA1, cervical carcinoma can be treated with simple large loop excision of the transformation zone (LLETZ), without further affecting fertility potential. Compared with this approach, hysterectomy has not been associated with improved survival rates if no lymph vascular space invasion and negative cancer margins are confirmed [10]. This approach can be applied in micro-invasive squamous cell carcinoma as well as adenocarcinoma, with similar outcomes [11].

In patients affected by cervical cancer at FIGO stages IA2–IB1 who wish to preserve fertility, radical trachelectomy with pelvic lymphadenectomy (confirming negative lymph node status) is the treatment of choice [12, 13]. Radical trachelectomy was first described by Dargent in 1994 [14] and it represents the most established surgical procedure for fertility preservation in women. The procedure has been reported for the treatment of squamous cell carcinomas and adenocarcinomas, with similar outcomes [15]. As operative techniques, vaginal, laparascopic, abdominal and robot-assisted trachelectomy have been described [13]. Long-term oncologic outcomes of trachelectomy seem not to differ compared with radical hysterectomy, and a long-term survival rate of 98.4 % and a relapse rate of only 4.5 % have been reported [16, 17]. Perioperative complications have also been similar when compared with radical hysterectomy [13]. Further development of non-invasive nuclear methods to identify lymph nodes in patients with early-stage cervical cancer might improve future patient selection for this type of fertility-sparing surgery [18].

After trachelectomy, over 60 % of tissue samples have demonstrated absence of residual tumor [19]. Therefore, conization in combination with laparoscopic lymphadenectomy has also been described as an appropriate procedure in selected patients presenting with early-stage cervical cancer (FIGO IA2 and IB1) and tumors < 20 mm. Women thus treated have succeeded in conceiving in 47 % of cases and the 5-year disease free survival reported of 97 % [20, 21]. However, although data are promising, the number of cases published is still small and further research is needed to implement this technique as a clinical routine.

In cases of more advanced disease with a tumor size >2 cm, initial neoadjuvant chemotherapy followed by radical trachelectomy and lymphadenectomy has been suggested by some authors [22, 23]. This approach has been shown to correlate with high fertility rates and no differences in oncologic outcome compared with immediate trachelectomy without chemotherapy [24]. Because of few reported cases and no long-term follow-up outcomes, this procedure should still be regarded as experimental.

In selected cases where radiotherapy or chemoradiation are necessary, the ovaries can be protected by ovarian transposition to remove them from the radiation field [25–27]. However, depending on the radiation dose and radiation scatter, the efficacy of this procedure has been reported to be about 50 % [28, 29]. If assisted reproductive treatments involving IVF are needed thereafter, the ovaries are often difficult to access for ovum pickup. Ovarian stimulation in connection with subsequent cryopreservation of oocytes or embryos before cancer treatment is thus indicated in such cases [6, 30–34]. However, even if ovarian function is preserved, or oocytes or embryos have been cryopreserved, irradiation of the uterus may cause irreversible damage. Although cases of good obstetric outcome have been reported after fertility preservation among women with a heavily irradiated uterus [35], unsuccessful results should be expected and in many cases surrogacy will be necessary [36].

If oocyte or embryo cryopreservation are not feasible, the emerging technique of cryopreserving ovarian tissue for later retransplantation might serve as a viable tool to preserve fertility in some cancer patients. Heterotopic and orthotopic transplantation sites have been described, with resumption of ovarian function [35, 37–39]. Up to now more than 60 children have been born worldwide after ovarian tissue transplantation [39, 40].

In cases of ovarian tissue cryopreservation, concerns have been raised as regards the risk of reseeding cancer cells at time of retransplantation, if they are present in the tissue preserved. Ovarian metastasis has been reported in 6 % of patients with adenocarcinoma of the cervix and 1 % of patients with squamous cell carcinoma [41]. Nevertheless, of five published cases of retransplantation of ovarian tissue in women with previous cervical carcinoma, none have resulted in relapse [32, 37].

For women undergoing radical surgery with hysterectomy, the chance of childbearing is only possible by means of a womb transplant. Successful results have been obtained by a Swedish team that has led this project over many years [42] and these procedures are expected to extend to several US centers in the future [43].

Data on assisted reproductive treatments after cervical cancer are scarce. In one study a prevalence of infertility of 13.5 % among patients with previous vaginal trachelectomy was reported [44]. Of these, cervical factor infertility was found in about 40 % of cases, indicating a need for intrauterine insemination as the first-line treatment approach. In other series of cases reported, 80 % of women conceived after subsequent fertility treatments [44, 45].

Regarding quality of life, compared with women with radical hysterectomy who had at least one ovary, patients who had undergone trachelectomy had similar sexual satisfaction and quality of life after surgery [46]. However, another study group reported low sexual satisfaction in the first year after surgery compared with healthy subjects and patients after abdominal hysterectomy. However, this effect decreased over time and after one year these patients had similar sexual satisfaction (but with a persistently reduced QoL) when compared with healthy controls [47]. These results are consistent with those of another study reporting bladder-emptying problems in more than 40 % and lymphedema in more than 10 % of cases, reflecting a lower QoL in patients after vaginal or abdominal trachelectomy compared with healthy controls [48].

Cold-knife conization and laparoscopic lymphadenectomy, on the other hand, are not associated with reduced sexual satisfaction and quality of life [49].

### Endometrial cancer

Endometrial cancer comprises 7.1 % of all new cancer cases in females. In the US, 55,000 new cases of endometrial cancer cases were expected in 2015, with a median patient age of 62 years. Seven percent of endometrial cancer patients are under the age of 45 years [1, 2]. Patients at higher risk of presenting endometrial carcinoma are overweight women and those with polycystic ovarian syndrome (PCOS) [50]. The standard treatment of endometrial cancer involves hysterectomy and bilateral salpingo-oophorectomy, due to the hormonal sensitivity of endometrial tumors [51]. In endometrial cancer IA without infiltration to the myometrium and no extrauterine involvement, conservative treatment can be offered to women who wish to maintain fertility. To counsel a women wishing fertility-sparing treatment options properly, myometrial evaluation by MRI should be performed [52].

For women with early-stage endometrial cancer, treatments involving use of progesterone either orally (600 mg medroxyprogesterone acetate daily or 160 mg megestrol acetate daily) or delivered by an intrauterine device (levonorgestrel-releasing IUD) have been described. The combination of IUD and oral progesterone treatment has also been proposed [53]. In retrospective studies a 72 % positive response rate to treatment has been reported [54–56]. In prospective studies, the treatment of complex endometrial hyperplasia using a levonorgestrel IUD has been shown to achieve greater regression in histology, and lower relapse rates than treatment with oral progesterone [57–59]. Lower rates of hysterectomy have also been reported after treatment with levonorgestrel IUDs [57–59].

Generally, relapses are frequent and occur in up to 50 % of cases that undergo conservative treatment [60]. Standard conservative treatments should be followed-up by hysteroscopic examinations every third month and endometrial sampling [61]. In cases of recurrence a second cycle of progesterone treatment has been associated with response rates of up to 89 % [62].

The combination of surgical resection and progesterone treatment has been associated with good oncologic and pregnancy outcomes in a small number of patients (Table 1) [63].

In women free of relapse, pregnancy should be achieved within the shortest period of time, and assisted reproductive treatments may have a place in reducing time to conception, thus reducing the time at risk of recurrence. Ovarian stimulation in cases of endometrial cancer has been an issue because of the supraphysiological estrogen levels attained during hormone treatments required for recovery of oocytes for IVF, and possible tumor stimulation. A few cases of successful live-births after IVF in women with previous endometrial cancer have been reported [64–71]. In these patients infertility treatment was not associated with an increased cancer recurrence rate [72].

The addition of letrozole to standard gonadotropin protocols has been proposed for ovarian stimulation among women with estrogen-sensitive tumors [73, 74]. The protocols, initially developed for women with breast cancer, could also be used in patients with endometrial cancers [75]. The performance of ovarian stimulation with a levonorgestrel IUD in situ has also been found to minimize the effect of estrogenic stimulation on the endometrium [76].

Whenever the desired family size has been reached, patients should undergo hysterectomy and bilateral salpingo-oophorectomy as a result of the persistent relapse risk [77].

A proportion of women treated for cancer might achieve pregnancy by surrogacy agreement, which is the carrying of a pregnancy by a third party (surrogate), a procedure that is allowed in some countries. Surrogacy gives the possibility of having biologically related children if gametes have been previously cryopreserved [76, 78].

As regards the QoL of women who have undergone fertility-sparing treatments in connection with early-stage endometrial cancer, the results of a meta-analysis indicated improved outcomes after treatment with levonorgestrel IUDs compared with oral progesterone, with reduced weight gain, sleep disorders, headaches, mood and libido disorders [79, 80].

### Ovarian cancer

Ovarian cancers make up 2.6 % of all female cancers. In 2015 around 21 300 new cases of ovarian cancer were diagnosed in the US. The median age at diagnosis is 63 years, with 12 % of patients under the age of 44 years [1, 2]. The different approaches in fertility-sparing surgery in cases of ovarian cancer and borderline ovarian tumors are summarized in Table 2.

**Table 2:** Fertility-sparing interventions in women with borderline ovarian tumors or ovarian cancer

Diagnosis	Type of Surgery	Description	Reproductive and Obstetric Outcomes	Oncologic Outcome	Quality of Life
Borderline Ovarian Tumor FIGO Stage Ia	Unilateral oophorectomy/bilateral cystectomy	Removing the affected ovary only, keeping in place the unaffected one and the uterus	Spontaneous pregnancies have been reported with favorable obstetric outcome [99]	Higher recurrence rates in fertility-sparing surgery compared with radical surgery, with no difference in mortality [97, 98]. Recurrence 0 %–20 % versus 12 %–58 % when only cystectomy was performed [6]	High quality of life and higher sexual satisfaction scores after fertility-sparing surgery [103]
Borderline Ovarian Tumor FIGO Stages Ic–III	Unilateral oophorectomy/bilateral cystectomy, peritoneal staging, pelvic & para-aortic lymphadenectomy, omentectomy	Removing the affected ovary only, thorough oncological staging	Pregnancy rate of 86 %, more than half of the patients required fertility treatment [99]	No difference in recurrence or survival compared with radical surgery removing both ovaries and the uterus [6, 99].	Lack of data
Ovarian Epithelial Cancer FIGO Stage IA, grade 1	Unilateral oophorectomy, peritoneal staging, pelvic & para-aortic lymphadenectomy and omentectomy	Removing the affected ovary only, thorough oncological staging	Pregnancy rates of >60 % Pregnancies have been reported with favorable obstetric outcome [145]	5-year survival 87 %, recurrence 7–12 % [6, 17]	No difference in quality of life aspects or sexual satisfaction scores compared with radical surgery [46]
Ovarian Epithelial Cancer – FIGO Stage IA, grade 2–3 or Clear Cell Carcinoma	Unilateral oophorectomy, peritoneal staging, pelvic & para-aortic lymphadenectomy, omentectomy and adjuvant chemotherapy	Removing the affected ovary only, thorough oncological staging Adjuvant platinum-based chemotherapy	Pregnancy rate of 80 % with live-birth rate of 65 % in women presenting with cancer grades 1–3. Higher number of women with cancer grades 1–2 attempting pregnancy in comparison with women with grade 3 cancers [87]	No difference in recurrence or survival compared with radical surgery [86]	Lack of data
Malignant Germ Cell Cancers grade I	Unilateral oophorectomy, peritoneal staging, omentectomy, pelvic & para-aortic lymphadenectomy and adjuvant chemotherapy	Removing the affected ovary only, adjuvant BEP chemotherapy has been recommended, or expectant management	76 % pregnancy rate. Pregnancies have been reported with favorable obstetric outcome [147, 148]	Fertility-sparing surgery has not been associated with impaired oncological outcome [108]	Good quality of life reported with good psychological health and sexual function [129]

Modified from: Rodriguez-Wallberg KA, Oktay K. Fertility preservation during cancer treatment: clinical guidelines. Cancer management and research. 2014;6:105-17

Abbreviations: *FIGO* International Federation of Gynecology and Obstetrics, *BEP* bleomycin, etoposide and cisplatin

#### Epithelial ovarian cancer

Most cases of epithelial ovarian cancer are diagnosed at an advanced stage, making it the most lethal tumor of all gynecological malignancies. Standard treatment consists of bilateral salpingo-oophorectomy, hysterectomy, omentectomy as well as pelvic and para-aortic lymphadenectomy [81].

In women presenting with epithelial ovarian cancer diagnosed at an early stage (typically FIGO stage IA) who wish to preserve fertility, unilateral salpingo-oophorectomy together with appropriate staging, omentectomy, pelvic and para-aortic lymphadenectomy can be performed to preserve the uterus and one healthy ovary [82]. If the contralateral ovary appears macroscopically normal, most authors discourage sampling of it due to impairment of ovarian reserve and causation of additional adhesions by performing the biopsies [82]. In cases of epithelial ovarian cancer with bilateral ovarian involvement a conservative approach should not be applied [83].

Laparoscopic fertility-sparing surgery has been shown to be a feasible approach in cancers of FIGO stage IA and the 3-year survival rate is about 95 % [84]. In patients presenting with a higher-risk early-stage ovarian cancer (IAG3 or higher) some authors have described fertility-sparing procedures in connection with non-impaired survival rates. However, the level of evidence regarding fertility-sparing surgery in high-risk ovarian cancer is limited due to the very small number of cases published [85]. In one study, if recurrence after fertility-sparing surgery occurred, long-term survival was 87 % as regards ovarian and 48 % as regards extra-ovarian relapse [82]. Data are still insufficient as regards other tumor types such as clear-cell carcinoma, but no differences in survival rates after fertility-sparing surgery have been reported in these patients when compared with women who have undergone radical surgery or fertility-sparing surgery in connection with non-clear-cell carcinoma [86]. Overall 5-year survival rates have been reported to be as high as 87 %, with approximately 12 % of patients suffering cancer recurrence after fertility-sparing surgery, when combining both low- and high-risk cancers [17].

The use of platinum-based adjuvant chemotherapy has been proposed for patients with high-risk ovarian cancer (IAG2 or higher) as well as clear-cell carcinoma after fertility-sparing surgery [85, 87].

Some authors have suggested the addition of assisted reproductive techniques using gonadotropic ovarian stimulation for egg retrieval after the performance of fertility-sparing unilateral oophorectomy. These procedures are aimed at safeguarding fertility potential by cryopreservation of embryos or oocytes for the future. The patient, thereafter, may undergo adnexectomy of the remnant ovary in a subsequent operation [88]. Reduced ovarian reserve may be a concern in women with previous ovarian operations [89]. Up to now, data are lacking on ovarian cancer relapse rates after gonadotropic stimulation. However, data on women at high risk of ovarian cancer as a result of BRCA mutations are reassuring and no association between gonadotropic ovarian stimulation and ovarian cancer has been observed in these patients [74, 90].

Ovarian tissue cryopreservation in patients with early ovarian cancers or borderline tumors is highly controversial but has been described by some authors [32]. As autotransplantation of the retrieved ovarian tissue is not feasible due to the risk of reintroducing malignant cells, alternatives have been discussed, such as culture and maturation of oocytes gained from the tissue in vitro, a procedure still under development which could be used in the future [91].

As regards QoL, available data indicate no major differences in sexual satisfaction or sexual concerns in women who have undergone fertility-sparing surgery compared with women who have undergone radical surgery [46].

#### Borderline ovarian tumors

Borderline ovarian tumors (BOTs) comprise 10–20 % of ovarian epithelial tumors [92]. In one study, among patients younger than 40 years, one third of ovarian cancer cases had borderline ovarian tumors [92]. Survival rates are about 99 %, with 70-month disease-free survival in cases of stage I tumors, and the survival rate in cases of stage III tumors is about 89 % [3].

Because of relatively young age and good prognosis of the disease, conservative surgery can be performed in most BOT patients. Usually, adnexectomy on the affected side is performed, since cystectomy of the tumor has been associated with higher recurrence rates [3]. In case of bilateral BOTs, unilateral adnexectomy and contralateral cystectomy can be performed in women who wish to maintain reproductive potential. Even though fertility outcomes are uncertain, oncological prognoses similar to those of patients treated by means of radical surgery have been described [93, 94]. Surgical staging and histological subtypes (micropapillary and stromal micro-invasion) of BOTs have had no impact on recurrence [95]. Although associated with good prognosis overall, a higher level of lethal recurrence has been reported in cases of micopapillary serous BOT [96].

In general, conservative treatment of BOTs is associated with higher recurrence rates compared with radical treatment [3, 97]. However, after a follow-up period of seven years mortality has been reported to be very low and most authors regard conservative surgery as safe [98]. In a recent study on 59 patients concerning the role of fertility-sparing surgery in cases of advanced borderline tumors (FIGO stages IC–FIGO III) it was concluded that fertility-sparing surgery was not associated with relapse or mortality [99].

After conservative treatment of BOTs, patients should be counseled about the risk of diminished ovarian reserve following repeated conservative ovarian surgery or adnexectomy, and fertility counseling should be provided. Oocyte cryopreservation for future use can be an option for many of those women who do not have the intention to attempt pregnancy in the short term [89]. Due to the limited amount of data available it is not clear whether ovarian stimulation affects relapse time [100, 101]. In in vitro models no detrimental stimulatory effects of FSH or estradiol (E2) were found in BOT cells [102].

After suffering BOTs patients report a good quality of life and good sexual function. Fertility-sparing surgery is not associated with a higher QoL, but patients after such surgery showed higher-level sexual activity than patients treated radically [103].

#### Germ cell tumors

Malignant ovarian germ cell tumors are rare (3–5 % of ovarian tumors), but are the most common ovarian tumors in very young women (< 20 years of age) [104, 105].

The majority of patients with malignant ovarian germ cell tumors are diagnosed with stage 1 disease as a result of the rapidly growing character of this kind of tumor [105]. Overall survival rates in cases of germ cell tumors are encouraging and fertility-sparing surgery is not associated with worsening of outcome [106–108].

Ovarian germ cell tumors are relatively heterogeneous, and there is great variation in management. In cases of immature teratoma, 5-year survival rates at stages I and II have been described as being as high as > 93 %, with higher recurrence rates in cases of grade 2–3 tumors and advanced-stage tumors [109, 110]. In yolk-sac tumors after fertility-sparing surgery and standard neoadjuvant chemotherapy 5-year survival has been found to be > 90 % and a fertility-sparing approach has been suggested irrespective of cancer stage [111–113]. In pure dysgerminoma, 10-year disease-free survival was > 90 %, with overall survival around 100 % [114, 115]. Due to this excellent long-term outcome, several authors suggest fertility-sparing treatment at all stages of ovarian dysgerminoma [116]. The usual treatment of ovarian germ cell tumors consists of unilateral adnexectomy, peritoneal staging and omentectomy [117]. However, some authors have described less invasive surgical procedures involving unilateral adnexectomy, cytology and peritoneal sampling in cases of dysgerminoma and immature teratoma limited to the ovary [110, 114]. In yolk-sac tumors, however, complete staging has been associated with a favorable outcome as a result of different adjuvant treatment at advanced stages [111, 113].

Bilateral disease is uncommon in cases of germ cell tumors and if the contralateral ovary appears macroscopically normal no biopsy is advised owing to the risk of extra adhesions and impairment of ovarian reserve [118, 119]. After fertility-sparing surgery, chemotherapy with bleomycin, etoposide and cisplatin (BEP) has been associated with improved disease-free survival [120]. However, recently a surveillance approach has been suggested for 50 % of patients with early stage I tumors [121]. In cases of early-stage yolk-sac tumors (stage I) surgery patients (with chemotherapy limited only to cases of relapse) showed higher recurrence rates but no difference in overall survival, saving 77 % of patients from chemotherapy [122]. However, in cases of higher-stage yolk-sac tumors standard-dose BEP chemotherapy has been associated with favorable overall survival rates and no apparent compromise of fertility rates [113, 123]. In early-stage pure dysgerminoma also, chemotherapy is only recommended in cases of relapse, according to several authors [114, 115]. In patients with immature ovarian teratoma, stage I, grade 2–3, adjuvant chemotherapy has been recommended by some authors, while the results of several studies suggest that an expectant approach with chemotherapy only in relapse situations in these patients may be more appropriate [124–126]. This is important because germ-cell cancer survivors treated with chemotherapy have shown relatively high chemotherapy-related secondary malignancy rates later in life [127]. Reproductive function, on the other hand, has been reported to be relatively good, with more than 80 % of patients retaining reproductive function after chemotherapy and surgery [116, 119]. IVF treatment in patients after germ-cell tumor therapy has been described in only a few cases [128].

Overall quality of life scores in germ-cell tumor survivors are good, with fertility preservation playing an important part [129].

### Reproductive outcomes after fertility-sparing surgery in cases of gynecological cancer

Gestation in women treated for gynecological cancer may require specialized surveillance, in particular if the treatment has resulted in anatomic disturbance of the cervix or the uterus due to operative procedures or radiation therapy.

In one study, in cases of early-stage cervical cancer, after LLETZ resection no impairment of fertility was observed. However, depending on the depth of resection, patients showed a higher risk of preterm delivery (OR 1.7) and premature rupture of the membranes (OR 2.69), with no effect on neonatal outcome [130]. Many spontaneous conceptions have been reported after radical trachelectomy, with rates of over 80 % when robot-assisted trachelectomy was performed [131]. Pregnancy rates of up to 60 % have been reported after abdominal and vaginal trachelectomy [132, 133]. Interestingly, even after neoadjuvant chemotherapy and vaginal trachelectomy in cases of IB1 cancer, a pregnancy rate of 86 % has been reported in women who attempted to conceive [134]. Performance of assisted reproductive techniques to achieve pregnancy has been reported to be necessary in up to 50 % of cases [135].

In general, rates of pregnancy loss after trachelectomy are higher than in the general population [133]. As a result of amputation of the cervix, high risks of preterm delivery and premature rupture of the membranes have been described [136, 137]. Recent data indicate relatively favorable outcomes, with more than 90 % of patients delivering in the third trimester [131]. However, the data are conflicting and whereas one study group reported 65 % of infants prematurely born (< 37 weeks) but only 4 % at less than 32 weeks of gestation [44], a recent review reported 28 % of premature children born before the 32nd week of gestation in a large population of > 300 live-births after trachelectomy [17]. Routine performance of cerclage is still a matter of controversy, and cerclage-related complications have been described [133, 138]. Several studies describe higher delivery rates achieved at term or during the third trimester after cerclage [139, 140]. In any case, the risk of prematurity should be considered as well as access to a center with specialized neonatal care. Frequent vaginal ultrasonography should be performed to assess the risk of prematurity associated with shortness of the cervix, and fetal lung maturation should be induced when necessary [141].

As a result of postoperative scar tissue after trachelectomy, elective cesarean section after 37 weeks of gestation is recommended [136].

Pregnancies in women with previous endometrial cancer have been reported, with success rates of over 60 % in women who attempted pregnancy [63, 72]. Pregnancy is achieved faster after treatments involving assisted reproductive techniques and miscarriage rates seem to be comparable to those in the general population [72, 142]. Overall, no adverse outcomes related to cancer treatment have been observed in over 75 live-births reported after endometrial cancer [17, 72].

In women with fertility-sparing surgery for ovarian cancer, use of assisted reproductive treatments involving IVF is indicated in many cases owing to a reduced ovarian reserve after repeated ovarian surgery or unilateral adnexectomy [143]. In series of cases reported, over 60 % of women who actively attempted pregnancy conceived, and miscarriage rates were low (< 30 %) [144, 145]. At present more than 220 pregnancies after ovarian cancer have been reported, with an overall miscarriage rate of 17 % [17]. Similar data have been reported for women treated for BOTs [99]. Interestingly, adjuvant chemotherapy has not been associated with infertility, but young age at the time of chemotherapy has been associated with premature menopause later in life [145, 146]. After fertility-sparing surgery in connection with germ-cell tumors, 76 % of patients who sought pregnancy conceived naturally [147], and pregnancies in patients after fertility-sparing surgery and germ-cell tumor treatment did not show any complications [148].

International guidelines for fertility preservation have been published and access to fertility preservation for young female cancer patients encouraged, in particular by use of assisted reproductive methods globally available and regarded as clinical routines, such as cryopreservation of oocytes or embryos after emergency IVF [6, 30–33, 149]. As regards fertility-sparing surgery for fertility preservation among women with gynecological cancer, global utilization of the methods available is currently unknown. In a recent European study, data was collected from several countries, demonstrating a low incidence of fertility-preserving surgery and it raised concerns as regards the need to centralize such treatments at accredited units, to ensure a sufficient number of patients at each center, with maintenance of good healthcare quality [150].

### Risk-reducing salpingo-oophorectomy in women at high risk of ovarian cancer who wish to preserve fertility

Women who are carriers of BRCA1 mutations present with a 39–46 % lifetime risk of developing ovarian cancer, and for carriers of BRCA2 mutations the lifetime risk is 12–20 %. The ovarian cancers that predominantly develop in BRCA1 and BRCA2 mutation-carriers are of serous or endometrioid histology and of high grade. In women with known BRCA mutations, periodic screening for ovarian cancer by way of assay of CA125 and transvaginal ultrasonography is recommended after the age of 30–35 years, or 5–10 years before the youngest age at which ovarian cancer was first diagnosed in the family [151].

Risk-reducing salpingo-oophorectomy has been shown to reduce the risk of ovarian cancer by 85–90 % and it should be offered to women with a BRCA mutation by age 40, or after the conclusion of childbearing [151].

Women who are carriers of BRCA mutations may have not yet built their families at the recommended age of risk-reducing salpingo-oophorectomy, and some of them may wish to undergo procedures to preserve fertility. Data on fertility preservation for BRCA mutation-carriers are largely linked to their concomitant risk of breast cancer, and in that respect reports are reassuring, as the ovarian stimulation and IVF procedures required to cryopreserve embryos or oocytes have not been shown to negatively affect the risks of breast cancer or breast cancer relapse in reported patient series [74, 90, 152]. Although pregnancy appears to be safe for BRCA mutation-carriers after breast cancer, specific studies on women with BRCA mutations are lacking. Such women may elect to utilize preimplantation genetic diagnosis during IVF to avoid transmitting the mutation to their children, but this option may create additional psychological distress, and, therefore, thorough counseling and psychosocial evaluation are essential [153]. Additionally, it has been noted that carriers of BRCA mutations may have lower ovarian reserves and can experience earlier menopause than non-mutation carriers, and thus the reproductive span of BRCA carriers may be 2–4 years shorter than that in the general population [74, 154].

## Conclusions

Current data on fertility-preservation options for women with early-stage gynecological cancer indicate oncological safety and high efficacy of fertility-sparing surgery. Some women presenting with sub-fertility may need to undergo assisted reproductive treatments to achieve pregnancy, which has not been shown to affect the oncologic outcome negatively. International guidelines for fertility preservation have been published and these underline the importance of timely discussion of the impact of cancer treatment on future fertility, and options for fertility preservation in all patients of reproductive age. The role of fertility-sparing treatment at more advanced stages of gynecological cancer has to be analyzed in further studies as a result of scarce data in this field.

## Abbreviations

BEP, bleomycin, etoposide and cisplatin; BOT, borderline ovarian tumor; BRCA, breast cancer 1/2 mutation; E2, estradiol; FSH, follicle-stimulating hormone; IUD, intrauterine device; IVF, in vitro fertilization; LLETZ, large loop excision of the transformation zone; OR, odds ratio; PCOS, polycystic ovary syndrome; QoL, quality of life
